# A comparison of deep-learning-based inpainting techniques for experimental X-ray scattering

**DOI:** 10.1107/S1600576722007105

**Published:** 2022-09-28

**Authors:** Tanny Chavez, Eric J. Roberts, Petrus H. Zwart, Alexander Hexemer

**Affiliations:** a Advanced Light Source, Lawrence Berkeley National Laboratory, Berkeley, CA 94720, USA; b Molecular Biophysics and Integrated Bioimaging Division, Lawrence Berkeley National Laboratory, Berkeley, CA 94720, USA; c Center for Advanced Mathematics for Energy Research Applications, Lawrence Berkeley National Laboratory, Berkeley, CA 94720, USA; d Berkeley Synchrotron Infrared Structural Biology Program, Lawrence Berkeley National Laboratory, Berkeley, CA 94720, USA; Argonne National Laboratory, USA

**Keywords:** X-ray scattering, image inpainting, deep learning, mixed-scale dense networks, tunable U-Nets

## Abstract

The implementation is proposed of image inpainting techniques for the reconstruction of gaps in experimental X-ray scattering data. The proposed methods use deep learning neural network architectures, such as convolutional autoencoders, tunable U-Nets, partial convolution neural networks and mixed-scale dense networks, to reconstruct the missing information in experimental scattering images. In particular, the recovered pixel intensities are evaluated against their corresponding ground-truth values using the mean absolute error and the correlation coefficient metrics. The results demonstrate that the proposed methods achieve better performance than traditional inpainting algorithms such as biharmonic functions. Overall, tunable U-Net and mixed-scale dense network architectures achieved the best reconstruction performance among all the tested algorithms, with correlation coefficient scores greater than 0.9980.

## Abstract 2

A number of machine-learning-based algorithms are presented for the reconstruction of gaps in experimental X-ray scattering images through inpainting approaches.

## 1. Introduction

X-ray scattering is a nondestructive technique commonly used for materials and structure characterization in different applications, such as the development of pharmaceuticals (Munjal & Suryanarayanan, 2021 ▸; Amaro & Mulholland, 2018 ▸; Maveyraud & Mourey, 2020 ▸), the analysis of myelin (Schulz *et al.*, 2020 ▸; Georgiadis *et al.*, 2021 ▸; Müller *et al.*, 2021 ▸) and amyloid structure (Liu *et al.*, 2016 ▸; Choi *et al.*, 2021 ▸) for disease detection in human brain tissue, the evaluation of human tissue nanostructure (Müller *et al.*, 2010 ▸; Georgiadis *et al.*, 2021 ▸) in biomedical imaging, the effects of cellular membrane thickness on intracellular transport (Heberle & Pabst, 2017 ▸; Khondker *et al.*, 2021 ▸), the inspection of skimmed milk nanostructures in food science (Yang *et al.*, 2021 ▸; Christiansen *et al.*, 2021 ▸), determining the stability and flexibility of polymer-like wires used in sub-nanometric materials design (Ni *et al.*, 2018 ▸; Liu & Wang, 2020 ▸), and many others.

A generic X-ray scattering experiment uses a 2D detector to collect the scattering data. Although this technique produces high-definition results, its experimental setup introduces image defects due to the experimental geometry, the beamstop and the detector inter-module gaps (Liu *et al.*, 2017 ▸). Thus, X-ray scattering images are commonly masked to remove the corrupted or missing pixels from the experimental results (Shimizu *et al.*, 2016 ▸; Li, 2021 ▸). Although the remaining pixels in the masked image present trustworthy information for the materials characterization of the sample, the presence of gaps introduces a pattern discontinuity that can affect the computational analysis of the samples. While the traditional processing methodology of scattering or diffracting data for deriving structural information does not and should not require estimates of gap-masked data (Pande *et al.*, 2018 ▸; Ashiotis *et al.*, 2015 ▸), certain processes, such as automated sample-type identification (Liu *et al.*, 2019 ▸) and initial orientation determination in single-particle X-ray scattering (Bellisario *et al.*, 2022 ▸), are greatly assisted by gap-less data. Hence, the reconstruction of gaps within X-ray scattering images represents an important task for specific data analysis steps within an automated data analysis pipeline or self-driving instrument (Noack *et al.*, 2021 ▸).

Image inpainting is a restoration process that estimates the content of missing regions within images and videos and has sparked special interest in the area of computer vision (Elharrouss *et al.*, 2020 ▸; Jam *et al.*, 2020 ▸). Conventional inpainting algorithms use well known mathematical and statistical approximation methods such as biharmonic functions (Damelin & Hoang, 2018 ▸), Cahn–Hilliard equations (Bertozzi *et al.*, 2006 ▸), anisotropic diffusion by partial differential equation modeling for propagating boundary data (Bertalmio *et al.*, 2000 ▸), low-rank tensor completion (Li *et al.*, 2020 ▸; Liu *et al.*, 2021 ▸) and Gauss–Markov random field priors (Satapathy & Sahay, 2021 ▸; Efros & Leung, 1999 ▸). Alternatively, machine learning (ML) approaches have potential for inpainting tasks. Among the most commonly used techniques, we mention general regression neural networks (Su *et al.*, 2021 ▸; Kanhar & Chandak, 2021 ▸), convolutional neural networks (CNNs) (Matsui & Ikehara, 2020 ▸; Jiang *et al.*, 2018 ▸; Liu *et al.*, 2018 ▸), generative adversarial networks (Chen, Zhang *et al.*, 2021 ▸; Zhao *et al.*, 2020 ▸) and two-stage generative models which refine coarse predictions with global contextual information (Pathak *et al.*, 2016 ▸; Yu *et al.*, 2018 ▸).

While deep-learning-based inpainting techniques have been widely applied for the reconstruction of facial and scenery features of images, their implementation in X-ray scattering data, particularly in experimental scattering data, has not been extensively explored in previous work. To the best of our knowledge, there exist just a handful of previous studies that aim to reconstruct the lost information in X-ray scattering data, generally accomplished by considering the symmetry of the structure of the data in a non-ML fashion or through ML approaches using simulated data (Liu *et al.*, 2017 ▸; Bellisario *et al.*, 2022 ▸). Since beamline scientists are currently using ML-based algorithms to process the large quantity of data they collect (Chen, Andrejevic *et al.*, 2021 ▸; Schwarz *et al.*, 2019 ▸), it is of great importance to reconstruct the missing regions in scattering images to avoid the introduction of distortion and bias into their post-processing analysis. Hence, this paper proposes the implementation of image inpainting techniques based on deep learning approaches.

Here we explore and optimize a number of popular convolutional neural network architectures to reconstruct the masked areas in scattering images. CNNs are considered a powerful feature extraction technique due to their simultaneously trained compression and reconstruction stages (Rumelhart & McClelland, 1987 ▸; Wickramasinghe *et al.*, 2021 ▸), allowing one, potentially, to identify the most relevant features within the masked input images for the reconstruction of the pixels located in the gap regions (Öztürk, 2020 ▸; Kornilov *et al.*, 2020 ▸; Liu *et al.*, 2018 ▸; Thakur & Paul, 2020 ▸). This work focuses on four neural network (NN) architectures: convolutional autoencoders, tunable U-Nets (TUNets), partial convolution NNs and mixed-scale dense networks (MSDNets).

The parameters of the network are optimized with respect to the *L*_1_ norm, as this provides a balanced trade-off between performance and complexity (Zhao *et al.*, 2017 ▸). Apart from the absolute error, the *L*_1_ metric, we report the Pearson correlation coefficient between the inpainted results and their corresponding ground-truth images. In addition, we further analyze the impact of inpainting within the context of latent-space estimation and how it differs from the masked and ground-truth latent spaces, respectively.

This paper is organized as follows. Section 2 describes the experimental setup that was used for the collection of the data set and the processing analysis, Section 3 introduces the inpainting algorithms, and Sections 4 and 5 present the experimental results and our conclusions, respectively.

## 2. Experimental setup

The data set used in this paper contains 6497 X-ray scattering images of size 1475 × 1679 pixels. The data are a random subset of a library housing over 30 000 scattering images of user data from a variety of transmission and grazing-incidence experiments collected on the Advanced Light Source SAXS/WAXS beamline 7.3.3 over the past decade (Hexemer *et al.*, 2010 ▸). The data comprise a large variety of materials, covering crystalline powders, liquid crystals, noncrystalline systems and thin films. The data were recorded on a PILATUS3 2M detector and collected at a constant X-ray energy of 10 keV with an *E*/δ*E* of 100. The PILATUS3 2M consists of three columns of eight vertically stacked individual detectors for a total of 24 detector modules. Two vertical gaps measuring 7 pixels wide separate the three columns of modules, while seven horizontal gaps measuring 17 pixels tall separate the eight rows. In total, the seven horizontal and two vertical gaps measure 17 × 1679 and 1475 × 5 pixels, respectively, with each pixel measuring 0.172 mm in length.

To collect the data obstructed by the horizontal gaps, the detector was exposed at an initial position, then moved vertically to collect the missing data under the same exposure conditions. While the vertical motion of the detector could technically introduce some small differences between these images, this is highly unlikely due to the resolution of the detector, which is less than 5 µm. Additionally, we followed the usual precautions to check for beam damage, which included moving the sample by the width of the beam when possible, and discarding images where heterogeneity could lead to different scattering events. The missing gap information of the initial image was then replaced by the data provided in that location from the second exposure. Averaging the common areas among these images was avoided because it leads to different noise statistics across the image, which makes fitting and interpretation more difficult. In addition, missing detector data due to dead pixels were also replaced where possible.

**Figure 1:**
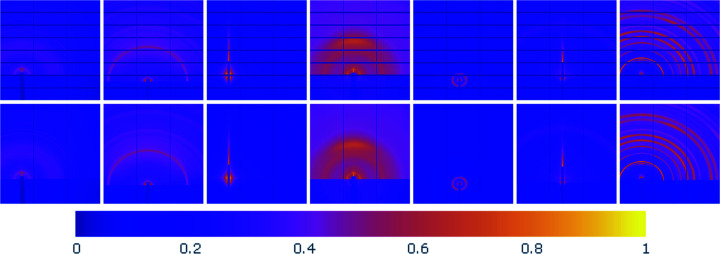
A data set overview, displaying the masked data in the top row and the corresponding ground truth in the bottom row. Vertical gaps are persistent in the ground-truth images.

Fig. 1 ▸ presents a sample of the data, where the first row contains the masked images and the second row contains their corresponding ground truth with the missing horizontal gap information filled in. However, the missing information in the vertical gaps persists. Although the pattern definition is visually clearer in the ground-truth images, the remaining vertical gaps continue to disrupt the pattern continuity and can negatively impact its post-processing analysis. Hence, it is of great interest to estimate the missing information within the entire gap grid (horizontal and vertical), even though the ground-truth images lack the pixel intensities along the vertical gaps. This paper tackles these two inpainting tasks simultaneously using the following data processing steps.

### 2.1. Pre-processing

Before applying the inpainting algorithms, the X-ray scattering images were resized to 512 × 512 pixels using a bicubic interpolation to reduce the computational complexity of the training operation. Even though this downsampling process reduces the resolution of the images, the goal of this study is to estimate the masked areas of X-ray scattering images to automate processes such as sample-type identification and initial orientation determination, which do not require the full-dimension image.

The resized images were then normalized and a logarithm was applied to highlight the weaker scattering areas. Additionally, the data were split into two subsets, where 4641 images were used for training and the remaining 1856 images were used for testing.

We highlight that the area to be inpainted, also referred to as the inpainting mask, does not vary in shape or location across the horizontal gaps because we employed a single detector to collect the X-ray scattering images presented in this study. While this mask does not have to be static for the correct operation of the inpainting algorithms, further training may be necessary for alternative inpainting masks, such as the masked areas of a PILATUS 300k detector.

**Figure 2:**
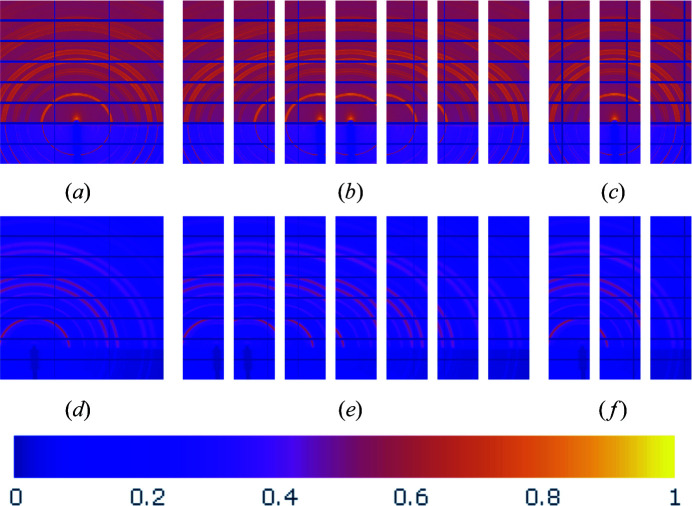
The data augmentation process. (*a*) Training image No. 541. (*b*) Image No. 541 cropped with the *qlty* package in Python, where the sections that contain vertical gaps are discarded due to lack of ground-truth information. (*c*) Augmented images obtained from training image No. 541. (*d*) Training image No. 143. (*e*) Cropped image No. 143. (*f*) Augmented images obtained from training image No. 143.

While the horizontal gap information missing from the masked (or input) images exists in the ground truth, inpainting the missing information from the persisting vertical gaps presents a greater challenge, as no ground-truth information exists to train against. To alleviate this challenge, we introduced a new set of training data consisting of smaller images extracted from the original data, further augmented with artificial vertical gaps placed over known data, effectively providing a target ground truth to train against. We illustrate the full gap grid data augmentation process in Fig. 2 ▸ for two training images and describe the steps below.

(i) Each masked training image is cropped into seven overlapping pieces of size 512 × 128 pixels with an overlap of 64 pixels along the horizontal axis, displayed in Figs. 2 ▸(*b*) and 2 ▸(*e*). Cropping is performed using the *qlty* Python package (Zwart, 2021 ▸), which will later be used for stitching together the cropped subimages that have been passed through the trained networks.

(ii) The cropped images containing vertical gaps are discarded.

(iii) Artificial vertical gaps are randomly introduced in the three remaining cropped images. The locations of these gaps are randomly assigned to the following horizontal coordinates: 

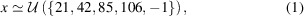

where −1 corresponds to no artificial gap added. These coordinates are chosen such that the artificial gaps in Figs. 2 ▸(*c*) and 2 ▸(*f*) are consistent in location with the four images with true gaps in Figs. 2 ▸(*b*) and 2 ▸(*e*).

Note that further reduction of the masked image size (below 512 pixels) resulted in smaller cropped images, which did not perform correctly in the neural networks that were tested in this work. Alternatively, applications that require training on the full-dimensional data set can potentially crop the input images following a process similar to the one described above in order to comply with the computational resources constraint. Additionally, this pre-processing of the 4641 training images results in a total of 13 923 augmented images for training.

### 2.2. Post-processing

While the augmented set of training images splits the original data into smaller image triplets, and replaces true vertical gaps lacking a ground truth with artificial gaps and a corresponding ground truth, the inference stage is performed differently. Namely, each testing image to be inpainted is cropped into the seven overlapping pieces seen in Figs. 2 ▸(*b*) and 2 ▸(*e*), described in Step (i) of Section 2.1. Each of the seven subimages passes through the trained network and all are stitched together after network inference. We note the importance of the artificial gap placement in the training data, as these gaps are consistent with the true gaps in the testing subimages. To recombine the seven overlapping subimages back into one single inpainted image, we once again use the *qlty* Python package, this time to perform a simple averaging of the overlapped areas. This overlapping helps alleviate the edge effects common in patch-based learning (Innamorati *et al.*, 2019 ▸; Cui *et al.*, 2019 ▸) shown by similar overlap averaging techniques (Pielawski & Wählby, 2020 ▸).

While the previously described process inpaints the testing data set in full, it does not provide areas to evaluate the quantitative performance of the inpainting algorithms along the vertical gaps due to the lack of ground-truth information. Therefore, these results are exclusively used for the quantitative evaluation of the inpainted areas along the horizontal gaps. To further analyze the vertical gaps, we perform a second inference test, where the testing data set undergoes the same data augmentation process described in Section 2.1 to generate ground-truth information artificially across these areas and use it for quantitative evaluation.

## 3. Inpainting algorithms

This section introduces the five inpainting algorithms studied in this paper, four of which are based on convolutional neural networks. The three algorithms using convolutional autoencoders, mixed-scale dense networks and TUNets enable blind inpainting in which the gap localization mask is not part of the input layer. The remaining two algorithms using partial convolutional layers and biharmonic functions require the masked-gap pixel locations as input.

### 3.1. Convolutional neural networks

A CNN is a shared-weight feed-forward architecture made up of several connected convolutional layers (Fukushima & Miyake, 1982 ▸; LeCun *et al.*, 1998 ▸; Goodfellow *et al.*, 2016 ▸) that approximates an underlying mapping function from input data to some ground-truth target domain, in this case the mapping of images missing the horizontal and vertical gap information to those containing the ground-truth horizontal and augmented vertical gap information. Each convolutional layer convolves the preceding layer’s output with multiple (typically several hundred) two-dimensional convolutional filters, or kernels. These filters are square matrices, typically of size 3 × 3 or 5 × 5, whose entries consist of weights to be learned during the network training and optimization process. Contrasted with earlier, more traditional, fully connected neural networks (Rosenblatt, 1958 ▸) in which a learnable weight (and learnable bias term) is assigned to *each* possible pair of nodes between neighboring fully connected layers (Xu *et al.*, 2019 ▸) (upwards of several tens of thousands per layer, depending on the number of pixels in the input image), CNNs enforce a more localized learning of image features and require *far fewer* weights to learn, resulting in deeper CNN architectures with more targeted learning. More intuitively, each filter acts as a smaller receptive field of view whose learned weights help identify different features within the image, beginning with lower-level features such as edges and boundaries consisting of lines and short curves in the early network layers, and progressing to more complex patterns such as faces and objects further along the network topology. Finally, the convolution between layer input and filters results in an intermediate feature map to be used as the next layer’s input, but not before being passed through additional nonlinear activation, pooling and normalization layers to help hierarchically decompose the input and probe for higher-level features.

#### 3.1.1. Convolutional autoencoder

**Figure 3:**
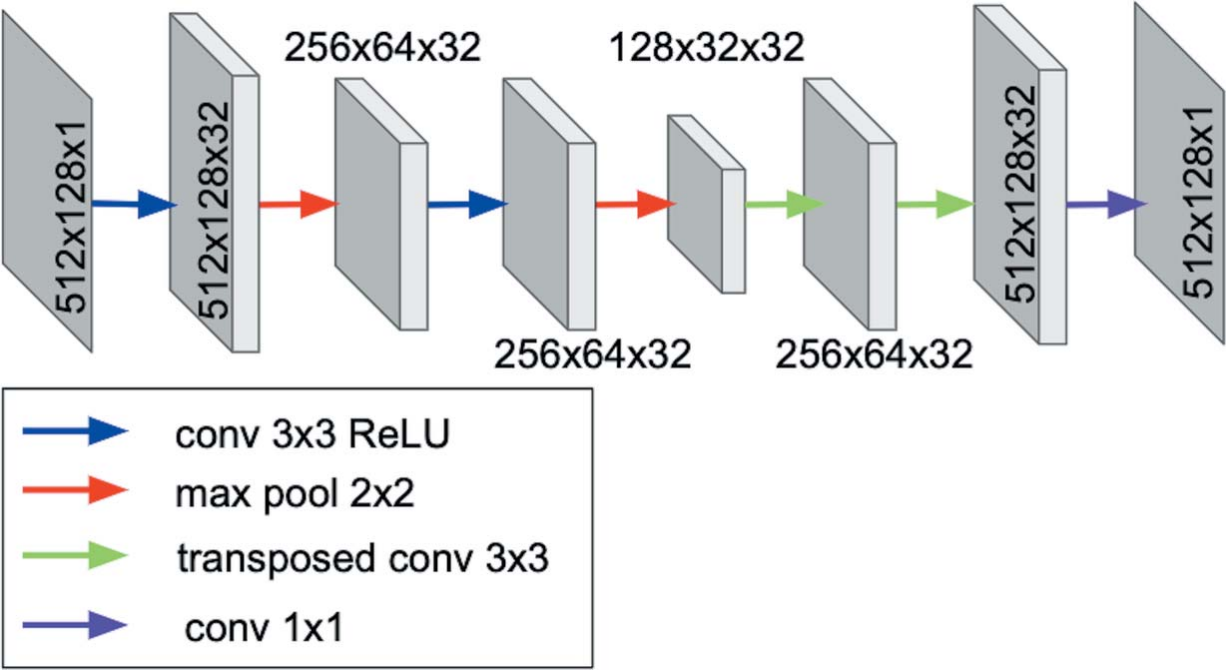
A schematic diagram of a two-layer convolutional autoencoder with 32 initial base channels.

The first convolutional inpainting approach corresponds to a CNN structure with a symmetric encoder–decoder architecture (Thakur & Paul, 2020 ▸; LeCun *et al.*, 1998 ▸). While relatively simple in structure, this method uses convolutional layers and max-pooling operations to exploit the feature extraction properties in the beginning encoder half of its architecture. As shown in Fig. 3 ▸, the encoder captures contextual information by compressing the input image into a lower-dimensional space of features, often referred to as the latent-space representation of the input, via max-pooling operations between adjacent convolutional layers. This informational ‘bottleneck’ forces the network to learn the most important feature. The second half of the network, the decoder, uses alternating convolutional and transposed convolutional layers (i) to reconstruct the non-gapped information and (ii) to inpaint the gapped portions of the images from the latent-space features. In both network halves, convolutional neural layers derive feature maps from the previous layer output by applying filters with a certain kernel size.

#### 3.1.2. Tunable U-Net

The U-Net architecture was first used in the segmentation of biomedical images (Ronneberger *et al.*, 2015 ▸). U-Nets extend the deep fully convolutional neural network architecture that was used for pixel-by-pixel classification (Long *et al.*, 2015 ▸) by introducing matching pairs of contractive and expansive operations that mirror each other at the far ends of the network. Similar to the convolutional autoencoder introduced above, the U-Net is a symmetric encoder–decoder system that first captures contextual information in the contractive encoder half, made up of stacked 3 × 3 convolutions, rectified linear unit (ReLU) activation and max-pooling operations, resulting in a ‘bottleneck’ of learned features that is similar to the autoencoder’s but much less compressed. Following this contractive phase is the up­sampling half which projects learned features back into higher-resolution image space to predict a pixel-by-pixel semantic segmentation. This decoder consists of layers of stacked operations similar to that of the encoder half: 3 × 3 convolutions, ReLU activation, skip connections via channel-wise concatenation and spatial dimension-doubling up-convolutions. Overall, contextual information is more readily propagated through the network by means of (i) the large increase in the number of convolutional channels over traditional fully convolutional neural networks, and (ii) long-reaching skip connections in the form of channel-wise concatenations of intermediate layer output between adjacent layers in the contracting and expanding halves. The skip connections effectively decouple the encoder and decoder halves, setting them apart from the convolutional autoencoders in which the two halves may operate independently of each other.

Many current denoising and segmentation applications default to U-Nets due to their simplicity and robustness (Çiçek *et al.*, 2016 ▸; Zhou *et al.*, 2018 ▸, 2020 ▸). In particular, deep learning frameworks deploying U-Net architecture backbones have shown potential for inpainting purposes (Öztürk, 2020 ▸; Kornilov *et al.*, 2020 ▸). For instance, the usage of consecutive convolutional filters within each layer maps the input images to a set of fundamental features that will later be employed for reconstruction of the missing pixel information. Similarly, the successive max-pooling of the data samples different scale spaces of the image, allowing local features to be more easily correlated with behavior and context at larger length scales (Noh *et al.*, 2019 ▸). Additionally, its overlap tile strategy is convenient for contextualizing prior localized information that was lost during the encoding phase of the NN. This strategy, in conjunction with the large number of feature channels in the decoding section, provides enough context to reconstruct the gaps in the later layers of the NN. Similar to the convolutional autoencoder architecture, U-Nets blindly inpaint the input image without the localization mask of the missing information.

**Figure 4:**
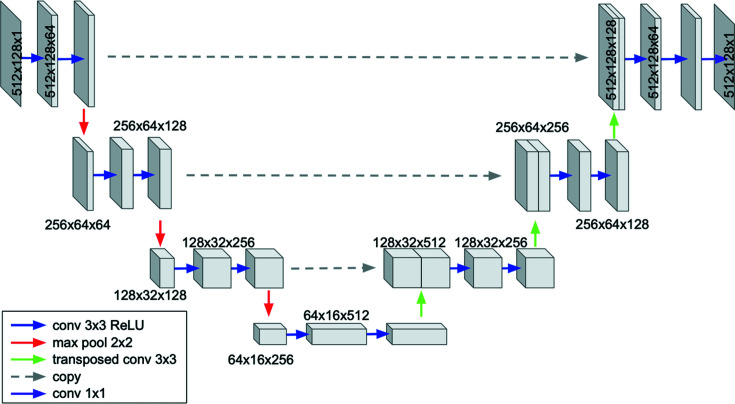
A schematic diagram of a four-layer TUNet with 64 initial base channels and a growth rate of 2.

The U-Net models used here, also referred to as TUNets, have been enhanced by allowing the specification of network-architecture-defining hyperparameters, such as the network depth, the initial number of channels and their growth rate after image contraction operators. This level of user-defined custom implementation was accomplished through the Python-based *pyMSDtorch* deep learning software library (https://pymsdtorch.readthedocs.io/), which allows easy tuning of the network hyperparameters to optimize its performance. The TUNet architecture and the associated hyperparameters used in this paper are shown in Fig. 4 ▸.

#### 3.1.3. Partial convolution neural network

**Figure 5:**
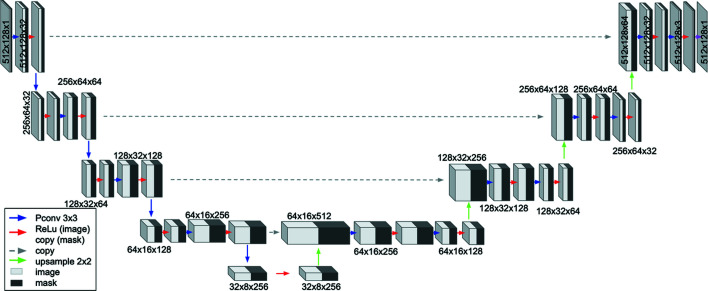
A schematic diagram of a five-layer partial convolution NN with 32 initial base channels and a structure similar to U-Net.

The third algorithm implemented in this paper corresponds to an alternative modification of a U-Net architecture, which replaces the conventional convolutional layers with partial convolutional layers, similarly to the approach used by Liu *et al.* (2018 ▸) and Thakur & Paul (2020 ▸). These modified layers take the gap localization masks as an additional input and perform segmentation-aware convolutions (Harley *et al.*, 2017 ▸) consisting of a masked convolution operation on the non-gap pixels and a renormalization of the convolutional output. Finally, for each forward pass, the network generates an update to the gap mask which aims to shrink the gap regions. Unlike the other proposed inpainting approaches, this method is not a blind inpainting approach since it takes into consideration the gap locations within the reconstruction process of the images. One major advantage of this implementation relies on its exclusive consideration of non-gap pixels in the convolution steps, which prevents the introduction of missing or corrupted pixel information during the training phase of the NN. The partial convolution architecture used in this paper is shown in Fig. 5 ▸.

#### 3.1.4. Mixed-scale dense networks

**Figure 6:**
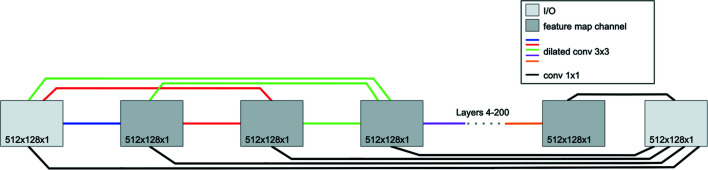
A schematic diagram of a 200-layer MSDNet with maximum dilation 15.

While U-net architectures remain popular, common implementations often require upwards of several million trainable parameters. This can lead to overfitting problems and harm network robustness, especially in applications where the quantity of training data is low (Goodfellow *et al.*, 2016 ▸; Srivastava *et al.*, 2014 ▸). In response, the MSDNet (Pelt & Sethian, 2018 ▸; Pelt *et al.*, 2018 ▸) architecture was developed as a deep learning framework containing *fewer* trainable parameters (typically two to three orders of magnitude fewer) than U-Nets. This is accomplished by densely connecting *all* network layers to encourage maximum reusability of image features and by replacing the typical scaling operations found in encoder–decoder networks with dilated convolutions (Yu & Koltun, 2015 ▸) in order to probe images at different length scales. By assigning a specific dilation to each MSDNet layer, the network can learn which dilation combinations are most effective. As a result, the number of network layers and the maximum integer dilation to cycle through are the two most significant hyperparameters to toggle, drastically simplifying network design. Additionally, the dense connections among intermediate feature maps create skip connections of *all* possible lengths. Lost spatial information is more readily recovered with the inclusion of these dense skip connections, which furthermore helps alleviate the vanishing gradient problem that plagues deep networks (Ioffe & Szegedy, 2015 ▸). The general MSDNet architecture is shown in Fig. 6 ▸, while the custom MSNet models used in this paper were deployed using the *pyMSDtorch* deep learning software suite.

### 3.2. Biharmonic functions

Biharmonic inpainting methods consider the inpainting procedure as a smooth surface extension problem leveraging numerical approximations of the planar biharmonic functions, solutions to the square of the fourth-order partial differential equation Laplacian operator which typically governs flows of viscous, incompressible fluids (Damelin & Hoang, 2018 ▸). In this context, the known pixel values on the boundaries of missing gaps act as boundary conditions for biharmonic function solutions, diffusing the known boundary values toward the gap center. This method is similar to the partial convolutional neural networks in that inpainting is performed inward from a gap boundary and it is not a blind approach, as inpainting requires the use of a gap location mask.

## 4. Experimental results

This section summarizes the inpainting results obtained using the proposed methods described in the previous section. A hyperparameter sweep consisting of nine TUNets and five MSDNets was performed for this article and is detailed in Appendix *A* [App appa]. Tables 2 and 3 show, respectively, the best performing TUNet (model 2) and MSDNet (model 3) that were used in the images shown in this article. A baseline performance metric was obtained using a well known inpainting algorithm based on biharmonic functions (Damelin & Hoang, 2018 ▸).

### 4.1. Training and evaluation metrics

To gauge the differences between ground-truth and model predictions of real horizontal gaps and artificial vertical gaps, we choose to minimize the *L*_1_ loss metric, as it remains a popular regularizer for various inpainting tasks (Liu *et al.*, 2018 ▸; Yu *et al.*, 2018 ▸; Chen, Zhang *et al.*, 2021 ▸; Yu *et al.*, 2021 ▸) and generally results in less blurring of inpainted regions (Isola *et al.*, 2017 ▸). Note that the *L*_1_ metric comprises the entirety of the loss function; no adversarial loss or additional regularization was used. Selected training rounds using the *L*_2_ loss metric resulted in less favorable inpainting of the gaps, both horizontal and vertical, in line with past reports (Zhao *et al.*, 2017 ▸).

For evaluation purposes, we report both the *L*_1_ metric and the Pearson correlation coefficient between the inpainted pixels and their ground-truth information. These metrics evaluate the inpainted results from different perspectives, where the *L*_1_ metric analyzes the magnitude of the prediction errors, and the correlation coefficient evaluates the strength of the linear relationship between predicted and ground-truth pixel intensities.

### 4.2. Training details

The *ADAM* optimizer (Kingma & Ba, 2014 ▸) was used to update the various neural network weights according to the *L*_1_ loss terrain. As for the learning rates, all neural networks were trained according to the same scheduler: an initial learning rate of 10^−3^ that dropped twice by a factor of 10 every 100 epochs for a total of 300 epochs. An exception was made for the 200 layer MSDNet in which the learning rate dropped every 60 epochs for a total of 180. Lastly, TUNet and 100-layer MSDNet training was performed on a single Nvidia RTX 3090 GPU with 24 GB memory capacity and 936 GB s^−1^ bandwidth. The remaining algorithms were trained on a single Nvidia A100 GPU with 40 GB capacity and 1555 GB s^−1^ bandwidth with a 32-thread AMD EPYC 7302 CPU.

### 4.3. Results

**Figure 7:**
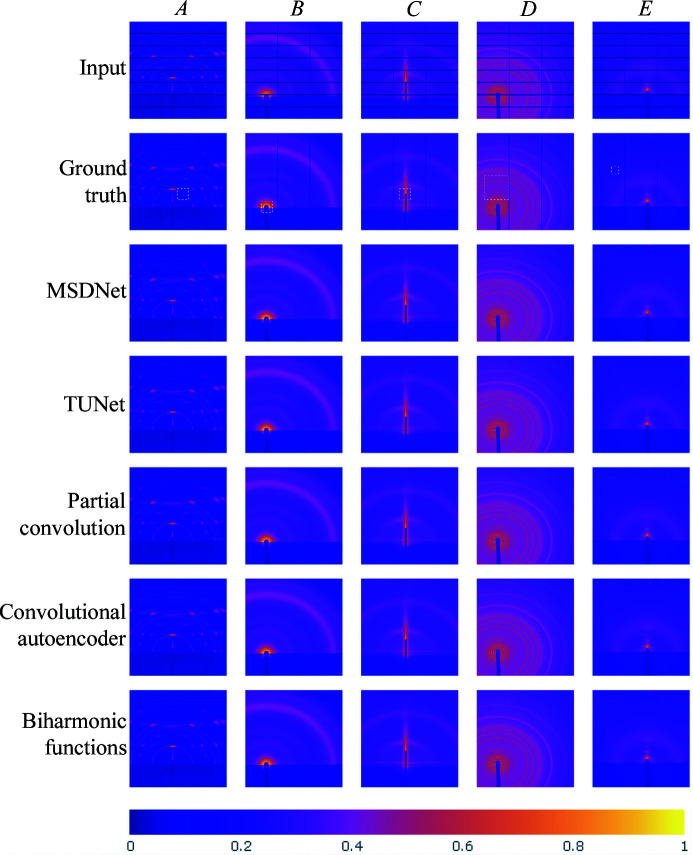
A comparison of inpainting methods, where the masked input data are displayed in the top row, followed by their corresponding ground-truth images in the next row. The dashed areas in the ground-truth images indicate the location of the close-up images presented in Fig. 8. The following rows present the inpainted results obtained from the deep learning methods studied in this paper, where the non-gap regions have been replaced with the original pixel intensities from the input images. These results are organized according to the overall performance per method from the highest to the lowest, as follows: MSDNet, TUNet, partial convolution, convolutional autoencoder and biharmonic functions.

Fig. 7 ▸ compares the qualitative performance of the inpainting methods by presenting five X-ray scattering images in our testing data set. We emphasize that the non-gap regions within the inpainted images have been replaced by the original pixel intensities from the input images. Hence, this replacement process avoids the introduction of predicted pixels outside the non-gap areas. The selected results correspond to test image Nos. 1326, 1474, 721, 442 and 337, which are referred to as samples *A*–*E*, respectively, in Figs. 7 ▸ and 8 ▸.

**Figure 8:**
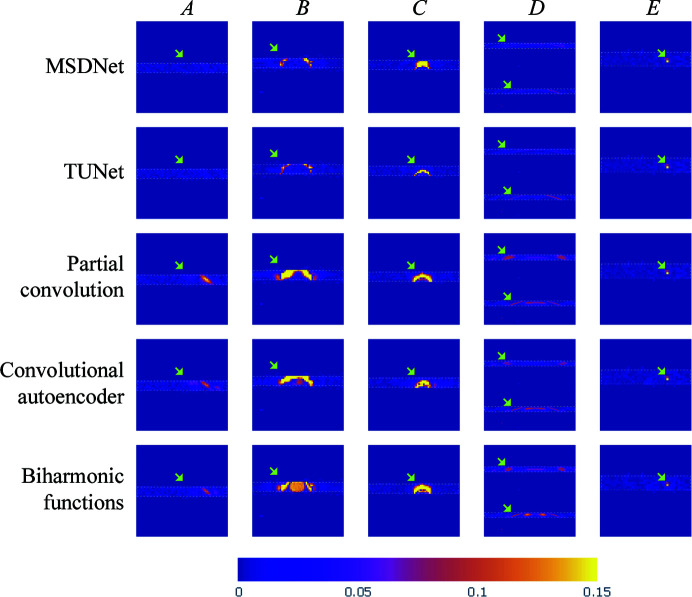
A close-up comparison of inpainting methods using difference maps between the ground truth and each inpainting approach, where the white dashed areas correspond to the boundaries of the horizontal gaps. To standardize the color map across all samples, the maximum value in the color bar has been set to 0.15, which means that the bright-yellow areas in these plots represent a difference of 0.15 or higher.

While most of the algorithms successfully reconstruct the majority of the missing pixels in the gaps, we can observe some pattern distortion and intensity contrast mismatch in a few images. Thus, Fig. 8 ▸ presents cropped sections of the inpainting results for close-up analysis using difference maps between the ground truth and each corresponding algorithm. For instance, sample *A* exhibits a disruption along the inner ring when using the biharmonic function, convolutional autoencoder and partial convolution approaches. Similarly, samples *B* and *C* exhibit some distortion near the beamline stop for most inpainting methods, with TUNet and MSDNet presenting the fewest differences among the proposed algorithms. Moreover, sample *C* has a minor beam interruption when using partial convolution. Sample *D* exhibits some pattern interruption along its rings. In this case, both the outer and inner rings present some missing spots in all inpainting methods, with TUNet and MSDNet introducing the least amount of distortion. Ultimately, sample *E* represents a special case when inpainting X-ray scattering images with peaks. In this sample, the ground-truth image of sample *E* contains a peak that was completely masked in the input image. Not surprisingly, since the input image lacks any information to detect the presence of this peak, none of the inpainting methods were able to recover it. This is bound to be a considerable limitation of inpainting, where fully masked features with no neighboring indicators cannot be accurately reconstructed.

**Figure 9:**
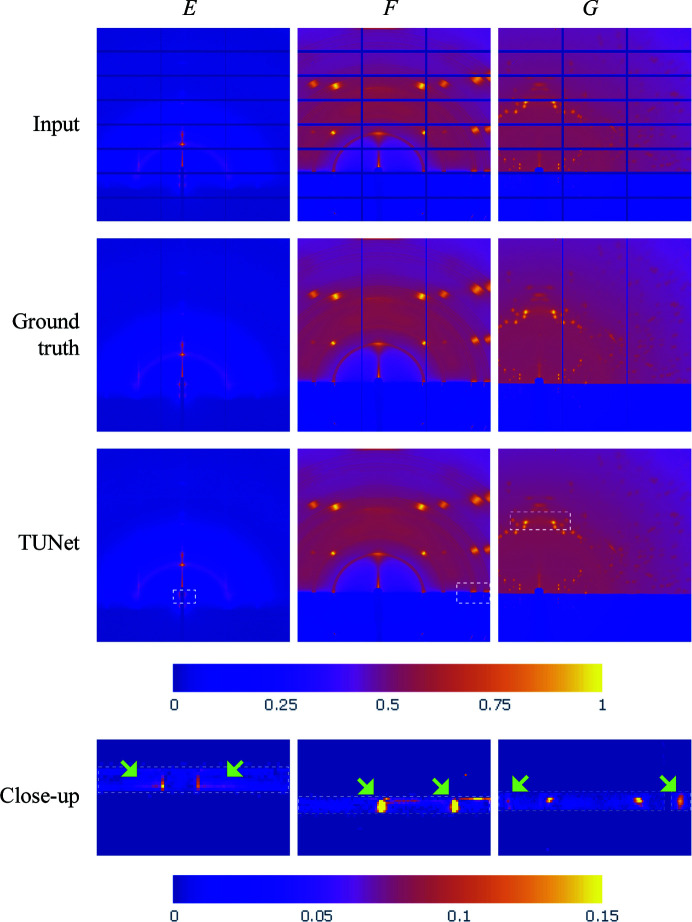
The qualitative performance of inpainting in X-ray scattering images with fully masked features, such as peaks. TUNet was selected as the inpainting reference, where the white dashed areas indicate the location of the close-up images at the bottom of the figure. The close-up images use difference maps to highlight the differences between the inpainted results and their corresponding ground truth, where the dashed areas correspond to the location of the horizontal gaps. To standardize the color map across all samples, the maximum value in the color bar has been set to 0.15, which means that the bright-yellow areas in these plots represent a difference of 0.15 or higher.

In order to discuss further the limitations of the proposed algorithms within X-ray scattering applications, Fig. 9 ▸ introduces three additional samples (*E*–*G*). Given the outstanding results obtained through TUNet, we have chosen this architecture as the inpainting reference for this analysis. While we can observe that the inpainted results for these images closely resemble their respective ground truth, we have selected an interest area in each sample that presents interesting feature cases. For instance, sample *E* contains a Yoneda peak across the sixth horizontal gap that is completely masked in its input image. As shown in the difference map, this peak could not be recovered by the inpainting approach due to the lack of neighboring feature indicators located in the non-gap areas of this image. Since most of the proposed algorithms make use of convolutional layers to reconstruct the missing areas of the input image, the information in the vicinity of the gaps is crucial for pixel intensity estimation performed by the set of filters. Similarly, sample *F* exhibits three peaks along the beamstop that were partially masked in the input image. Although these peaks were not fully masked, we can observe that TUNet could not reconstruct these features within the difference map close-up. The reason behind this behavior is the boundary location of the features, where the inpainting algorithm mistakenly reconstructed the horizon of the sample a few pixels above its correct location. Additionally, sample *G* presents a fourfold symmetry case with a number of masked peaks in its input image. The close-up area in the inpainted results presents four peaks along the third horizontal gap, where we observe that the peak on the left was better reconstructed than the one on the right of the image. Even though symmetry is an important feature in X-ray scattering applications, the inpainting algorithms proposed in this paper do not make use of this feature within their reconstruction models, mainly because the images have been cropped during the training stage of the inpainting process.

To quantify the performance of the algorithms across the testing data set, this paper uses the *L*_1_ error and the correlation coefficient between the predicted pixels and their ground-truth counterparts as the evaluation metrics of the inpainted results. In the horizontal gap analysis, we evaluate the pixels located across the horizontal gaps within the stitched images obtained by the *qlty* Python package, as described in Section 2.2. Unlike the horizontal gaps, the vertical gaps in our testing set lack ground-truth information. Therefore, we analyze the performance of the predicted pixels in the vertical gaps by adding artificial gaps through a data augmentation process in the testing set, which is descibed in Section 2.2. Table 1 ▸ summarizes the *L*_1_ error and the correlation coefficient score for each inpainting method. As a reference, the average pixel intensities across the gap regions in the testing data set were 0.2496 and 0.2394 for horizontal and augmented vertical gaps, respectively. These results further corroborate that TUNet and MSDNet have the best overall performance, followed by the convolutional autoencoders, the partial convolution NN and biharmonic functions, respectively.

Ultimately, blind inpainting approaches, such as TUNet and MSDNet, benefit from the presence of static masks across the data set collected in this experimental setup. While the trained models have obtained outstanding results in scattering images recorded on a PILUTUS3 2M dectector, alternative setups with different gap distributions may require further training to adapt the proposed models. Additionally, experimental setups with irregular masks (*e.g.* due to custom-shaped beamstops) may benefit from the partial convolution neural network architecture due to its ability to reconstruct irregularly shaped gaps in alternative inpainting applications (Liu *et al.*, 2018 ▸).

### 4.4. Latent-space analysis

To demonstrate further the importance of inpainting in X-ray scattering applications, we compared the spatial distribution of the latent vectors estimated with and without inpainting. Three different latent spaces were estimated by training an autoencoder with the original masked images, their ground truth and the TUNet inpainted images, respectively. For fairness of comparison, we used the same network architecture and training parameters for the estimation processes of all the latent spaces with a final dimension size of 200, similar to the approach used by Guo *et al.* (2017 ▸) and Liang *et al.* (2021 ▸).

**Figure 10:**
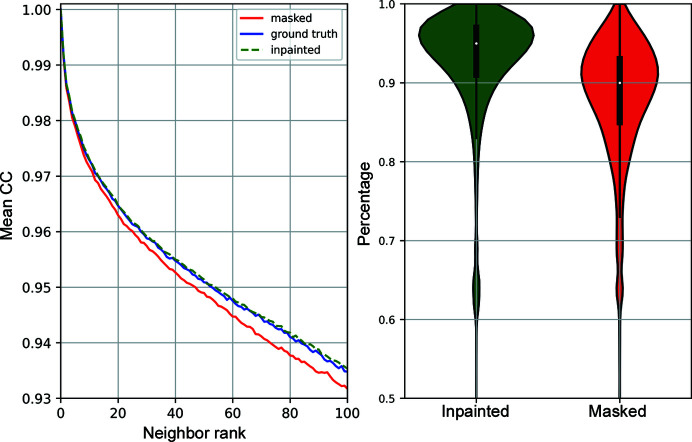
A summary of the latent-space analysis for masked, ground-truth and inpainted images. On the left, the figure presents the mean correlation coefficient as a function of the neighbor rank, which was defined through the masked, ground-truth and inpainted latent spaces, respectively. On the right, the violin plots represent the distribution of the percentage of overlapping images within the 100 nearest ground-truth neighbors using the masked and inpainted latent spaces, respectively.

Fig. 10 ▸ summarizes the results obtained from the latent-space exploration. The plots on the left represent the variation in the mean correlation coefficient as a function of the neighbor rank, which was calculated using the Euclidean distance between latent vectors. To avoid an offset in the correlation coefficient values due to the masked areas, these coefficients were calculated using the masked images for all cases. As shown in these plots, the mean correlation coefficients obtained through the ground-truth and inpainted ranks are very similar, whereas the masked rank presents lower mean correlation coefficients. Additionally, we identified the 100 nearest neighbors for each image within the ground-truth latent space and later used this as a reference to quantify the overlap among the 100 nearest neighbors in each image in both masked and inpainted latent spaces. In the right-hand graph of Fig 10 ▸, the distribution of these percentages is represented through violin plots, where we can clearly observe that the inpainted distribution has a higher mean and a tighter tail compared with its masked counterpart.

Overall, if we use autoencoders to compress the images, the relationships between neighboring images in latent space for the inpainted images are equivalent to those obtained from ground-truth data, while for non-inpainted images this relationship deteriorates. Therefore, these results demonstrate that inpainting can improve the estimation of the latent space, which is a key step to automating sample-type classification processes based on machine learning approaches.

## 5. Conclusions

The results presented in this paper demonstrate that the missing information in X-ray scattering data can be successfully recovered using inpainting approaches based on deep learning NN architectures. This study has proposed four architectures, namely convolutional autoencoders, TUNet, partial convolution NN and MSDNet, which were compared with a well known inpainting technique using biharmonic functions. Among these methods, TUNet and MSDNet achieved the best reconstruction performance with *L*_1_ errors of 4.4746 × 10^−3^ or lower, and correlation coefficients greater than 0.9980, for both horizontal and vertical gaps in the testing data set.

Unlike other inpainting challenges, X-ray scattering images present regular masks with rectangular shapes that remain static for all images. Hence, blind inpainting approaches similar to the TUNet and MSDNet algorithms presented in this work can effectively reconstruct the missing pixels within the scattering image. While these conditions are particular to this application, the experimental results prove that inpainting algorithms originally designed for image processing of facial and scenic features can potentially be used for the reconstruction of X-ray scattering data. Considering the promising latent-space results we have observed, our future work aims to analyze further the importance of inpainting for the pattern classification of X-ray scattering images through ML-based deep learning methods.

## Figures and Tables

**Table 1:** Quantitative analysis of testing results

		Biharmonic functions	Convolutional autoencoder	TUNet	Partial convolution	MSDNet
Horizontal gaps	*L*_1_ error (10^−3^)	6.6225	6.4807	4.4746	6.2228	4.3958
	Correlation coefficient score	0.9958	0.9964	0.9980	0.9958	0.9981
Vertical gaps	*L*_1_ error (10^−3^)	4.9841	5.4004	3.9240	4.9388	3.9288
	Correlation coefficient score	0.9972	0.9974	0.9986	0.9971	0.9986

**Table 2:** Summary of TUNet hyperparameter sweep Model 2 is the best performing network.

	Model								
Parameter	0	1	2	3	4	5	6	7	8
Depth	4	4	4	5	5	5	6	6	6
Base channels	16	32	64	16	32	64	16	32	64
Growth rate	2.5	2	2	2	2	1.5	2	2	1.5
Parameter count (millions)	0.535	2.140	8.556	2.158	8.630	6.033	8.648	34.586	34.512
Batch size	180	100	50	180	100	50	180	100	50
Training loss (10^−3^)	5.07	2.25	1.20	3.98	2.92	1.24	4.33	2.13	1.19
Validation loss (10^−3^)	5.15	2.25	1.23	4.01	2.94	1.31	4.39	2.40	1.28
Cross correlation score	0.9965	0.9991	0.9994	0.9983	0.9991	0.9993	0.9981	0.9994	0.9992

**Table 3:** Summary of MSD hyperparameter sweep Model 3 is the best performing network.

	Model				
Parameter	0	1	2	3	4
Layers	50	100	100	200	200
Maximum dilation	12	12	20	15	20
Parameter count (millions)	0.012	0.045	0.045	0.181	0.181
Batch size	30	7	7	6	6
Training loss (10^−3^)	1.78	1.54	1.61	1.15	1.16
Validation loss (10^−3^)	1.62	1.14	1.21	1.15	1.16
Cross correlation score	0.9990	0.9993	0.9991	0.9994	0.9994

## Appendix A Hyperparameter sweep

Considering that TUNet and MSDNet presented the best overall performance during our preliminary analysis, we performed a hyperparameter sweep to study further the inpainting performance of these two architectures on X-ray scattering images. For TUNets, the hyperparameters that were considered in this analysis were the depth of the network, the number of base channels, the growth rate and the batch size. MSDNets considered the number of layers, the maximum dilation of the convolutional layer and the batch size. Tables 2 ▸ and 3 ▸ summarize the hyperparameters used for these analyses with their corresponding training and validation metrics. The best performing networks, selected on the basis of the minimum training/validation losses and the highest cross correlation score, are model 2 for TUNet and model 3 for MSDNet.
